# Technologies of Trauma: Flesh Witnessing to Livestreaming Online

**DOI:** 10.1007/s42087-020-00120-y

**Published:** 2020-06-01

**Authors:** Yasmin Ibrahim

**Affiliations:** Digital Economy and Culture, Queen Mary University of London, Bancroft Building, Mile End, London, E1 4NS UK

**Keywords:** Trauma, Flesh Witnessing, Witnessing, Livestreaming, Digital Technologies, Memory

## Abstract

This paper examines the trajectory of witnessing and testimony through modernity, utilizing the notion of ‘technologies of trauma’. Such a notion acknowledges humanity’s reliance on technologies to transmute history, trauma, or memory, transforming technologies from medium and machine into artefacts for circulation and exchange. The transcendence of medium into artefacts as sites of trauma equally highlights the socio-political frames within which testimony is extracted and witnessing is enacted, unleashing trauma as a cultural form and a resonant genre of popular consumption. This paper, in tracing the trajectory of these technologies of trauma and its cultural turn from the sensorial to mass witnessing to the aesthetic regimes of immediacy in the digital age, comes to term with our machinic bind in releasing trauma and its aestheticization as a cultural artefact and the unethical challenges, which unfold in such a proposition. The development of print, photography, television, and digital platforms as technologies of trauma reiterates the popularization of trauma, witnessing and testimony as cultural forms reiterating how these are intimately implicated in our emergence as active consuming communities of trauma and in tandem how these remake us as vulnerable subjects through the circulation of trauma as a cultural form.

## Introduction

We live in a constructed world. A world of sounds, narratives, and images, which seize upon knowledge and power regimes to impress their authenticity over other competing constructions. In these assemblages of making and unmaking the world, the realm of witnessing and the means in which we express and solidify these as truth testimonial and expression of cultural trauma remain an intense arena of scholarly enquiry over time. Modernity itself emerges through the amplification of its ideals through literary and visual artefacts in time and space or in spreading libertarian ideals or competing economic imperatives through mediated technological forms as a mechanism to solidify ideals and ideologies in societies. In raising witnessing within the spectre of modernity and its enterprise with machines and technologies, our ability to express, record, and archive lapses into a culture of witnessing as a phenomenon inextricably bound with not just machines but also our moral codes, legal systems, values, religious beliefs, and equally our sense of social justice. Within the metanarratives of modernity, it coalesces with the emergence of trauma as a cultural form through social, economic, and political disruptions and disorientations within the global landscape and the cultural modes humanity appropriates to express trauma (see Smelser 2004; Sztompka 2000; Sztompka 2000a).

While an immense amount of scholarship has accrued on the notion of witnessing through technologies (Oliver 2009:Ibrahim 2020; Ebbrecht 2007), from the invention of print and photography to today’s digital economy, this paper acknowledges the significance of witnessing’s myriad functions in a modern society, examining the phenomenon of trauma as a cultural form through the lens of ‘trauma technologies’. In so doing, it traces the trajectory of witnessing in modernity from the premise of ‘flesh witnessing’ to the contemporary notion of ‘ruin porn’ in which the self is constructed through sites of trauma (Ibrahim 2015) and the notion of relentless sharing on social media sites in invoking the gaze of consuming communities online. In such a trajectory, the sensorial experience of flesh witnessing shifts to an aesthetic regime of communicating and curating the self through the ubiquity of digital imaging technologies from conflict zones. Examining mediated witnessing through the lens of ‘trauma technologies’, this paper argues that the exposition of human trauma and its projection, whether through human articulations or visual regimes, are prolifically bound with technologies. Technologies of trauma are more than recording devices; they amplify trauma through their technical bias; they bind trauma to the social and public spheres, igniting a culture of ‘traumatrope’ as a means in which to access and accelerate events, accord meaning, partake in them, or to be disenfranchised by their saturation through ‘compassion fatigue’ in mediascapes. In accelerated modernity the speed of mass media becomes a part of our lived reality, premising immediacy, instantaneity, and ubiquity. Virilio’s (2002) theory of accelerated modernity’s draws on the notion of communication technologies such as television and the internet abolishing time and space and unleashing a pace and speed to modern life, recomposing it through its frenetic activity and turbulence, and recalibrating it as a ‘mode of being’ defined through its ubiquitous and schizophrenic acceleration.

The paper utilizes the term ‘technologies of trauma’ to refer to the means by which technologies transmute trauma through their technical capacity and within their wider social and political processes, which bind these to our everyday lives in constructing our social realities. By recording, archiving, and re-playing trauma, these technologies can re-distribute trauma to new audiences, making these available ‘on-demand’ and through ‘click’ economies of downloading, co-creation, and curation in the digital economy. In enabling the release of testimony as an artefact, these technologies of trauma produce in renewed terms the audiences as ‘witnessing’ subjects. The coalescing of human trauma and its release through technologies has given rise to articulations of ‘witnesses in the media, witnessing by the media, and witnessing through the media’ (Frosh & Pinchevski 2008: 1). John Durham Peters describes witnessing as a sensory experience and witnesses as ‘the surrogate sense organs of the absent’ (Peters 2008: 25). Yet technologies of trauma are inadequate as machinic translators of the experiential. Technologies of trauma are instruments of projection, designed to circulate trauma and transform it into an ‘artefact for sharing’ from its originary form as personal solitary and embodied encounters. The ability to store and leak testimonies erect technologies of trauma as not only instrumental or ideological but disruptive entities. When deposited into our cultures of accelerated modernity these contain possibilities to move affective consuming communities or produce inertia, equally rupturing through disenfranchisement or unification through communion, without foreclosing their ability to swathe us with a renewed sense of vulnerability and anxieties.

By tracing how technologies have mediated trauma and testimony over time, this paper reviews the shifting terrain of witnessing as a cultural form (i.e., from flesh witnessing to the post-digital age), denoting a move from the sensorial to an aesthetic regime reiterating the predominance of the visual in new media platforms and its promiscuous bind with virality, in a networked digital economy which seeks human attention as a given. This paper argues that technologies of trauma in redistributing trauma through global communication architectures produce new subjects and subjectivities, which then pose renewed moral and ethical considerations in the age of the digital against the backdrop of accelerated modernity. This redistribution of trauma also situates modernity within a culture of trauma, inducing vulnerability as its spasmodic human condition where the human emerges as a fatigued form due to overexposure to this virtuality and virality of trauma. Technologies of trauma are then intimately implicated in the culture of witnessing and testimony production illuminating violence as configurative of modern society, and in tandem invoking humans through their vulnerability and their capacity to be the ‘wounded and wounding’ (Kristeva 2005) in human civilizations. The transcendence into the digital world represents an economy of ‘attention deficit’ combined with a saturation of ‘traumatropes’ online. Trauma as a digital form online is re-pressed through its instability of form where it can be converted to new formats, morphed and consumed without content, reiterating and intensifying anxieties about what we are called to witness online in view of diminished gatekeeping online and the ability to stream imagery and narratives both through the point of victims and perpetrators invoking pressing and unresolved ethical questions about ‘what we are made to witness and for whom?’

## Witnessing: From the Sensorial to the Aesthetic

The twentieth century has been described as the ‘age of change’ and is characterized through the speed, scope, depth, and wonder of changes driven by scientific and technological innovations (Sztompka 2000: 12). Drawing on Albert Camus, Virilio incisively points out that the twentieth century has been a pitiless century, it being the century of the Titanic, Chernobyl, Auschwitz, and Hiroshima (Virilio & Baj 2003: 67). The circulation of trauma as a cultural form has in tandem unfolded through time against this horrific chain of traumagenic events. While the etymology of the term ‘trauma’ from its Greek origins refers to a physical injury, it has now been extended to collective experiences of traumatized communities and beyond participants who are directly affected and to include psychological injury (Hałas, 2010: 315). Studies of hysteria and trauma have been major pre-occupations in modernity, veering beyond psychoanalysis to be received in other fields in recognizing the wider influence of cultural trauma as a condition of our modern lives (Smelser 2004; Jabarouti & Mani 2014: 159).

French historian Annette Wieviorka has referred to the present as the ‘era of the witness’ and testimony as a new genre-in-the-making that characterizes this epoch (see LaCapra 2009: 60). Witnessing, testimony, and trauma have an inextricable bind in modernity. Witnessing can be defined as an act of bearing witness, to testify, to give evidence, to be a spectator or an auditor of something, to be present as an observer, and to see with one’s own eye (Oliver 2003: 133). Within this ambit is the role of the subject and subjectivity of the witness. Corporeal or embodied witnessing is a highly subjective individual encounter revealing the vantage point of the witness as part of a bigger event, weaving in one’s affective and sensorial perception of that experience. As such the encounter emerging through one experiential state is amenable to contestation from others who have experienced the same event. Harari asserts, ‘By the sixteenth century it was already a common maxim that “of twenty who return from a battle, no two agree about the beginning, the middle, or the end, and recount it differently”’ (cf. Harari 2009: 216). As an embodied encounter of strife, the recounting of the narratives may convey their personal trauma but not entirely its sensory wholeness.

Harari (2009: 219) points out that ‘with time the transmittable nature of factual knowledge leads to eyewitnesses losing their authority to experts such as historians’. What is worth noting in Harari’s argument is the loss of authority over time to the historian where the expert takes over the re-narration of constructing history through a collective assemblage of witnessing narratives to mine the ‘facts’. Harari, 2009: 218–220) argues that in the early eighteenth century the emergence of a ‘culture of sensibility’ laid claim to the novel authority of ‘flesh witnessing’, which privileged personal experience rather than facts, signifying a shift in authority to the recounting of experience through the affective state of the witness. The poetics of the recounting was in its *contradictions* made distinct through one’s sensory experience and not judged in terms of their rank in the military and whose authority should preside in the recounting of history. The ‘fog of war’ as manifest in the different sensorial accounts emphasized the experiential as opposed to the factual in contrast to the earlier emphasis on the extrapolation of facts from individual encounters to form the collective history of war or conflict.

The authority of the witness as a form of outing the truth is equally about who has the voice to testify. George Haggerty (2003; 168), in writing on the taboo of sodomy in the eighteenth century, reveals the ‘keyhole testimony’ as one in which an observer watches certain sexual activities from a distance and then reports them to a listener or an audience, either in a court of law or another social situation, such as an anti-sodomy tract or a novel. Rather than get so close that the observer could touch the participants, a doorway or a partition usually separates the observer from the scene of sodomy and this precedence to a visual accounting of the scene offers a new set of erotic dynamics implicating the observe as a voyeur.

The poetic bind between fact and fiction, co-mingling with visual encounters means that the testimony is given over to techniques of envisioning the past and present. If witnessing can lapse into fiction to compose its narratives, the artefacts of the novel and literary texts have relied on history to narrate fiction. Jennifer Cooke (2009) explores how the plague, which devasted Europe in 1720, was reworked and re-imagined in works such as Daniel Defoe’s *A Journal of the Plague Year* (1722) and Albert Camus’ *The Plague* (1947). Cooke argues that literature can step into the gap between the official account and that of the eye-witness whose perspective is personal and therefore limited, and provide a narrative that gives the impression of having official and myriad eyewitness positions at its command. This witness, which fiction can provide, occupies a fantasy space. The work of literary texts as a mode of witnessing and narrating history is yet another rich area of cultural resource for human society.

Between fiction and fact mining, the genre of testimony as both an affective and critical practice often confronts the unspeakable. Research on Holocaust survivors highlights the problematic lapses in the chain of witnessing (Hennessy 2013:72). Agamben (1999) conveys this through the figure of the *Muselmann* in the death camps of the Holocaust. Straddling between being a corpse and being barely alive, the liminal figure represents the inadequacy of representation witnessing unspeakable horrors, which render it a living corpse. Allen Feldman (2004:163), in writing about biographical narrative as life history, oral history, and particularly those which are produced in the aftermath of ethnocidal, genocidal, colonial, and postcolonial violence, argues that such artefacts occur within specific structural conditions, cognitive constraints, and institutional norms. Writing with reference to the South African Truth and Reconciliation Commission, the question of who is human to be imbued with rights becomes an arena of both witnessing and the emergence of newly produced political subject and subjectivity through biographical testimonies. The biographical artefact emerging from political violence to be transacted as a literary artefact in the market or adduced as evidence in courts or other domains counts as ‘part and parcel of the cultural construction of human rights practices in our times’ and functions as a medium for historicity interposing itself between the witness, reader, auditor, adjudicator, and anamnesis’ (Feldman 2004: 163–164). Witnessing sets out to span social and epistemological distances, but sometimes they may not be bridgeable. The imaginary geographies that structure the flow of affect in these situations and the chasm that can be created across which empathic bonds simply do not cross (Hennessy 2013:72).

The early 1850s saw a major turning point with the introduction of new, cheaper, and faster technologies that opened up photography to commercialization on a vast scale, removing the medium from the exclusive preserve of scientists and specialists and ensuring its popularity (Green 2011: 91). This new visual terrain as a popular era of fixing testimony and temporal location of history was enabled through photography. Allowing new ways to visualize and consume the world beyond one’s own immediate space, photography produced a strange newness even with the known and familiar, while offering a means to fix and immobilize the external world (Green 2011). Photography as a technology was seen as producing a rupture from the previous world, imbuing the medium with magical and mysterious qualities and the conjoining of these qualities with witnessing meant that visual testimonials acquired a morbid interest within mainstream culture.

Beyond popular technologies that become embedded into our social constructions of reality and representation, artefacts of witnessing can move between the archive and history to be reposted into new cultural terrains. Caroline Wake (2013) points out that, ‘Video testimony has escaped the archive and arrived online, entering new visual and virtual domains’. Wake chronicles the Fortunoff Video Archive for Holocaust Testimonies, which was established in 1979 and which later moved to Yale University, set up with the objective of recording open-ended, free-flowing interviews (2013: 118). The video testimony as an artefact also attests to its mobility, finding itself on YouTube where it can encounter larger sets of disparate audiences consuming trauma and testimony enabling new modes of witnessing, which are spatio-temporally removed while harbouring an emotional co-presence.

Ellis (2000: 32) confers technologies of witnessing such as photography, sound-recording, film, and radio as expanding the realm of sensory witnessing. As technologies that distribute trauma and vulnerability, Ellis singles out television as a technology separated in distance yet united in time with this televisual co-presence, symbolizing the salience of both the witness and witnessing through its liveness and distribution of violence and trauma to a mass audience. This subjective realms of meaning-making as a witness in this virtual witnessing needs to equally reconcile with the production of the political subject in modernity and who can speak to these technologies of trauma. Feldman (2004), in speaking of the political subjection through modernity, invokes the work of Alain Touraine (1981) on social movements. From the agentic male bourgeois Habermas (1989) resurrects as the progenitor in the public sphere to the male proletariat in Marxist and anarchist theory as the agent of social change, the postcolonial sphere saw the emergence of new subjects positioned as witnesses in the discourses of violation of human rights. ‘Previously inadmissible social categories—women, ethnic and racial minorities, peasants, the colonized, sexual minorities, fauna and flora, the disabled and the diseased, youth and children—emerged as political agents with their own political agendas and diverse sites of political struggle’ (Feldman 2004). In tandem with this, Anne Cubilé (2005) lobbies to understand testimony within a broader framework as a performative act where the subjectivity of the witness and the phenomenon of witnessing is cognizant of the compromises and failures in enacting it, including the disparity between the political aspirations and historical limits of representing the other.

## Wound, Wounded, and Witnessing

Technologies of trauma exist within the interstice of what testimonies cannot quite capture. Kristeva’s (1982) accounting for the gap between bodies and words, and the fact that words are never quite enough to capture bodily experience, refers to this as a ‘wound’—locating it as a seat of our vulnerability where we are wounding and wounded because we occupy the space between bodies and meanings. Similarly, Felman and Laub (1992) argue that bearing witness to the ‘impossibility’ is also about the breakdown of witnessing. This wound, or what has in other scholarship been mapped as a ‘veracity gap’ between the experiential and the event, points both to the appropriation of witnessing into fields and modes of enquiry such as history and medicine with rationalist and empiricist traditions. For Kristeva, the constitutive part played by destructivity, vulnerability, and disequilibrium is integral to the identity of the human species and the singularity of the speaking subject (2005: 115). As such the notion of vulnerability needs to be co-located with other seminal notions of liberty, equality, and fraternity within the paradigm of Enlightenment humanism (Kristeva, 2005: 115). Vulnerability occupies a place ‘between being and meaning, between bodies and words’ and is reconfigured as forming the very basis of our vulnerability.

Kelly Oliver (2009:90), in writing about ‘technologies of violence’, invokes Judith Butler (2004) and Julia Kristeva in suggesting the need to accept and own our vulnerability, as disavowing is a moment of dismantling or undermining our democratic solidary leading into conflicts or war, particularly when we fantasize ourselves as invincible. The word ‘vulnerable’ originates from the Latin word *vulnerabilis* and in the Oxford English Dictionary is defined as ‘having power to wound; wounding’. A second meaning is those ‘that may be wounded; susceptible of receiving wounds or physical injury’. The notion of vulnerability, Oliver points out, is already configurative of violence, inducing the vulnerable to mean both wounding and wounded. In this sense, technology can reassemble the body within the Western social imaginary as both threatening and vulnerable, exploding in the screen, for example through the materiality of suicide bombers (2009: 90–91).

Oliver contends that human dependence on the natural world and other humans bears with it an obligation to conserve nature and ensure the justice of social relations, which sustain life. This entails the conscious experience of ourselves as subjects entailing a tension between one’s position as a subject and one’s subjectivity. Where the former is determined by history and politics, social world, and culture, the latter is a sense of agency constituted through interaction with others. As such, bearing witness calls upon our attachment with others and to identify with a collective cause or through social justice frameworks, reiterating our collective humanity. While the act of bearing witness can renew our social and collective bond with a wider humanity, it reassert us as subjects and ignites our sense of agency in acknowledging our subjectivity within a social world, reiterating our vulnerabilities and anxieties in the modern world.

The notion of wound and wounding then relates back to the idea of trauma in sociological theory, which draws from medicine and psychiatry. According to Sztompka, 2000a: 450), the discourse of trauma has been penetrating the domain of social sciences and humanities. Sztompka, drawing on Smelser (1963), theorizes cultural trauma through humanity’s disorientation and the upsets as well as pathologies induced through the disruptions caused by the structural tremors in society. Cultural trauma is then characterized through traumatic events, the modes in which a society may cope after an event or its post-traumatic adaptations within its cultural milieu. Cultural trauma can be evidenced through a propensity in culture to find artistic expression to transmute trauma, leading to cultural interpretation of events. Trauma, as a social fact (Durkheim 1982), means that groups can share a collective identity through trauma, which may both be imposed on them or be constraining. As such cultural trauma affects culture and us as subjects and, in tandem, our subjectivity.

Bearing witness involves a complex constellation of telling and not telling, disclosure and reticence, memory, truth, history, and fiction. (Davis 2018). Witnessing as located beyond the objective in what is supressed in terms of pain or trauma has been an area of increasing interest for the psychoanalyst as what is not articulated or how it is articulated becomes important in understanding the subjectivity of the witness. While historians were waiting to hear confirmation of what they already knew, psychoanalysts were listening to hear something new, something beyond comprehension (Oliver 2003: 139). The sensorial and affective nature of witnessing and its translation into artefacts have evolved through time with technological advances and a recognition that technologies can amplify trauma through their intrinsic technical bias. The receptivity to the sensorium of witnessing also increasingly premised the visual as a form of truth regime and enabled through media like television, which called on larger publics to become ‘virtual witnesses’ and to become political subjects through the act of televisual consumption. The screen and the obsession with the visual coalesce the sensorial with the visual, acknowledging screens as enabling haptic and affective encounters (Duncum 2002).

Bearing witness through art or the artefact itself present extreme ethical challenges to humanity. The tendency to collapse any distinction between the event and the experience for LaCapra (2009) produces a collapse of witnessing via the sublime and its excess. ‘There is insufficient differentiation and at times uncritical relation of an aesthetic to the sublime leading it to be reified within popular culture. The excess is an event which exceeds knowledge and representation yet demands a testimony’ (LaCapra 2009: 65). The collapsing of subject positions through the sharing of the experiential and creating a proximity with the witness through testimony is also about the problematics of secondary witnessing via technologies of trauma or artefacts. This he suggests marks a transfiguration of the ‘wound or trauma into the sublime’ (2009: 69). ‘As such the sacralization of trauma and the traumatic experience become intertwined in its configuration as the sublime. Trauma is projected as accompanied through the sublime through its elevation and exhilaration where it can be appropriated as a device to be misrepresented through its ideological re-fashioning. As such trauma becomes rebirthed through jouissance and the orgasmic or as limit-shattering experience. The enmeshing of the attraction with repulsion or is eroticization may equally produce resistance to enabling this promiscuity in art and artefacts’ (LaCapra 2009: 70–71).

Technologies of trauma do things. They are far from neutral and as objects that transmute trauma; they produce new subjects of witnessing and vulnerability of being human through virulent circulation and consumption of trauma. Occupying a perceptive and material realm, they reconstruct our social realities of an external world through witnessing and testimony. Within the realm of broadcasting and post-digital platforms they work with both the massification of virtual trauma and its re-commodification as entities for exchange on social media platform. The ability to produce and consume as well as publish online enables new modes of interactivity with trauma as artefacts online, where new modes of intimacy and proximity may emerge through interactive platforms and image curation in which visual content can be lifted and aestheticized devoid of context.

## Virtual Witnessing to Sharing Platforms

Elie Wiesel argues that broadcasting moving images turn modern citizens into witnesses of the events of their time, asserting that such technologies produce a new distinct form of perception, which ‘carries a sense of responsibility however weak towards those events’ and summed up as ‘they cannot say that they did not know’ (cf. Ellis 2008: 73). This distinct form of perception acquired through the televisual or the post-digital screen imposes new subjectivities on the consumers as witnessing subjects, invoking even in weak terms new modes of moral responsibility that by virtual witnessing one cannot claim to disavow through ‘not knowing’. As such, if witnessing as a phenomenon remains as an arena of scholarly enquiry, its production of subjects and subjectivity through technologies of trauma is a problematic terrain to manoeuvre. In inducing vulnerability and anxiety as a pervasive element of accelerated modernity through ‘disaster marathons’ (Blondheim and Liebes 2002) and dedicated satellite news channels, which broadcast traumatropes on a loop, the wound and wounding become entities of mass circulation.

Technologies of trauma seize this wound and amplify the vulnerability of the human condition. Redfield, writing on the circulatory power of the destruction of the Twin Towers and the Pentagon under the popularized idiom of ‘9/11’, chronicles how it became part of American cultural life.

The 9/11, mythologized as a popular chronotope of before and after, in fact conceals the existence of a ‘post-traumatic mood’ after the USA experienced or caused some of the catastrophes of the twentieth century, from the Great Depression, the Second World War, the atomic bombing of Hiroshima and Nagasaki, the Cold War, and the Vietnam War, which encompassed a range of human and economic catastrophes, which induced this mood (Farrell, 1998: 2). Redfield points out that despite the virulent circulation of 9/11 as a discourse and imagery on televisual platforms, these in fact represent a ‘blockage’. Invoking Dominick LaCapra’s (1998) reflections on historical trauma in the case of the Shoah, he points out that the hyperbolic commemorative efforts such as those on display in ‘9/11 discourse’ are in fact testimonials to blockage and as such make cultural trauma spectral and uncertain in terms of its public and private manifestations. He writes

The event called September 11 or 9/11 was as real as death, but its traumatic force seems nonetheless inseparable from a certain ghostliness, not just because the attacks did more than merely literal damage (that would be true of any event causing cultural trauma) but because the symbolic damage done seems spectral? not unreal by any means, but not simply “real” either (Redfield 2007: 56).

Redfield, in denoting this as ‘virtual trauma’, illuminates a violence inherent to all media technologies, which record and remember the unique only by effacing and forgetting it, to mark the violence produced through the violence of technologies. There is a risk to this usage in making one believe trauma is not entirely ‘real’ and may ironically work to ward off psychic trauma (Redfield 2007: 68). Technologies of vision as a mode of aesthetic regime offers its own logic of the ocular while producing truth and veracity through the visual and its ability to dwell on its repetition.

Spectacular violence as a form of testimony crosses boundaries between the real and reel, between history and fiction re-pressed through the voyeuristic and pornographic. Technologies, which transform into an artefact in the act of recording and disseminating testimonies of violence, through their ability to repeat and amplify, acquire a subjective and symbolic power. The re-machination of time and space through these technologies of trauma, and the repetition as well as mass circulation, produces disenfranchisement and degrees of wounding. Redfield (2007: 69) treats the violent moving imagery of 9/11 as ‘supplemental violation, an obscene repetition of injury’.

Trauma, pain, and testimony seize new formats in popular culture for others to consume in misery memoirs, daytime TV talk shows, and media representations of violence in general, but also by tourism to places of mass death (Rothe 2011:159–160). Berger (1980) in ‘Photographs of Agony’ refers to the audience’s sense of moral inadequacy in responding to atrocity photographs published in newspapers, raising the despair of inaction, on the one hand, and call to action through indignation, on the other. Rothe (2011: 160) points out that Berger’s notion indicates that depictions of atrocity give rise to a sense of moral inadequacy among the audience, based on their awareness of the vast discrepancy between the others’ suffering and their own comparative comfort. In addition, the obtuse removal of these depictions from everyday lives, according to Luc Boltanski (1999), presents fewer possibilities for action, turning trauma into a spectatorship.

The transcendence into a screen culture in the digital era has alluded to the human form entering a post-digital epoch of witnessing through the ubiquity of convergent technologies remaking the screen as a site of intersection between communication, communion, information, and entertainment processed through networked relationships and a complex assemblage of internet architecture. These coalesce human subjectivity with machinic logic, enabling audiences to interact in real time and transact themselves as commodities online through a profile culture and networking on social media sites. Screens attached to the body through smart technologies become an extension of the body and senses.

In referring to the screen as the site of post-human witnessing, Smith (2012) refers to ‘editation’ where the capacity to witness is proscribed through the digital architecture and platforms enacting post-humans as the new consuming entities. Post-humanism is understood as a temporal or epochal phase in which human capacities are perceived as extended through technological innovations and whose relations are dispersed across virtual networks (see Hayles 2008:135). For Myra Seaman (2007: 247), the post-human of popular culture ‘is a deliberately engineered form, a hyperreal entity imagined in the form breakthroughs, both fictional and real, in genetic manipulation, reproductive technologies, and virtual reality’. As a fused biotechnological product pulled across the fecund terrain of technological possibilities and as a test figure of new emergent immoralities posed by human advances in which the ethical constraints fail to keep up with the breakthroughs, the posthuman emerges as a disoriented figure. William J. Mitchell (2004) invokes this category as a composite of fleshware, hardware, and software underpinning it through its ‘hyper-individuality, hybrid materiality, and networked relationality’.

Through this ambit of the post-digital realm and the consuming figure of the post-human, we encounter yet more ethical and moral challenges in terms of witnessing, testimony, and the culture of trauma. Networked economies, virulent disseminating architectures, and algorithmic capital absorb content and re-distribute it through search engines, newsfeeds, content sharing sites, and networks. Witnessing through the platform economics of social networking sites, and degrees of trust which emerge though these relationships along with the lack of editorial oversight over newsfeeds in social networking sites, remediates witnessing and testimony extraction as an unstable terrain online. Testimonies of witnessing float within this architecture and swim without context abstracted from their origins. The flow of violent images and testimonies in the form of still and moving images online enact trauma as a part of this attention-hungry economy, inviting new forms of spectatorship with the availability of livestreaming and simulated environments in which (con)fusion prevails between the real and unreal, in view of digital formats having the capacity to be altered and remade.

Historical imagery and testimonies such as the Eichmann trial in sepia-tinged renditions vie with disparate contemporary content such as cat videos on image-sharing platforms with global reach. In this architecture of the attention economy and ‘soundbite’ society details are foregone for browsing and clicking through a vast repository of content personalized by machines understanding and predicating one’s consumption habits. The avalanche of content and speed of distribution reiterate the fatigued figure of the human as both producer and consumer or ‘prosumer’ who can bear witness and equally call on others to bear witness to her trauma through convergent platforms and smart technologies, which enable lay people to record and publish online. The digital as enabling simulated platforms, which recreate the reality of violence through digital games, absorbs social and political contexts to resurrect them as gameplay online. In such an economy, witnessing testimonies compete with ‘confessional cultures’, sensitive information leaking scandals such WikiLeaks and traumatropes igniting trauma as a resonant cultural form in the digital age. This instability of the digital manifested in its ability to shed its format, acquire new forms, be reproduced as a copy of a copy, or to simulate the real while subverting reality produces new vulnerabilities and anxieties. This also means we witness a complex digital architecture where trauma can become entertainment, disembedded from context, or curated through the aesthetic gaze of its audiences. For Edmund Burke both the sublime and the beautiful can be an assault on the senses and can strike the audience without a preparation, seizing the senses and imagination before one can understand it [107–08]. As such the profane and sacred can share a proximity as can the traumatic and aesthetic (Redfield 2007). Extracting pain and pleasure from trauma has elicited philosophers to acquire an ‘aesthetic distance’ such that pleasure does not thwart experiences of human suffering. Distance and proximity get re-mediated online in enabling the self to be recomposed through sites of trauma and for these to be commodified online.

The affordance of mobile and convergent technologies such as the smartphone or bodycams (i.e. recording cameras) embedded onto the body enable consumers to stream content live to image-sharing and social networking platforms online, making witnessing a live and present encounter. Visual content as unstable entities, which can be changed, morphed, memefied, and effaced in form and format, remakes the digital economy a conflicted site for witnessing. In addition, body-worn cameras and recording devices can enable audiences to witness from the vantage point of perpetrators in violent acts such as mass shootings, blurring the lines between reality, and the simulated violence of video games or the violence marathons of Hollywood productions. The centrality of visual media in mainstream culture and its appropriation by terrorist groups means that violent acts become artefacts for mass distribution online. In digital platforms the sacred and profane reside side by side and with the lack of consistent gatekeeping online, platform capital enacts its own moral governance of content producing violence through the modes in which it unleashes violent content to audiences en masse. Convergent recording/imaging and livestreaming facilities on social networking sites and video-sharing platforms mean that audiences can now be privy to the violence from the vantage point of the perpetrator, enacting mass shootings as entertainment which can be consumed and commented on in real time.

Kelly Oliver (2009: 99) points out that technology ‘both produces and reproduces the material and intellectual terrain of the contemporary landscape’. As vehicles which mediate our experience of an external world our corporeal and affective bodies become inextricably bound as ‘raw material’, as Oliver opines, with machinic instruments. Ranciere speaks about art having the ability to re-distribute senses. Similarly, technologies of trauma bound through modernity’s phenomenon of witnessing and trauma testimonials are bound with trauma as a cultural form reframed through the virulence of circulation in this platform economy. Technologies, which extract, archive, and redistribute trauma, can unleash new ethical challenges, and within the post-digital landscape it immerses us into new modalities of trauma consumption. Digital platforms, with their interactive architecture, induce new witnessing subjects defined by their capacity to re-distribute trauma and re-make trauma through the new media economy where content can be created, copied, reposted, and curated though personal archives. In the process, these architectures of interactivity and consumption equally produce new subjectivities through this ‘consuming’ witness.

## Conclusion

If, for the survivor, witnessing is a privileged or solitary experience, technologies of trauma extrapolate testimonies and project them onto consuming publics. The amplification of trauma through broadcasting technologies and the capacity inherent within these technologies for repetition construct trauma as a resonant cultural form in late modernity. Technologies of trauma hinge on the distribution of witness accounts, including real-time broadcasting which enable virtual and mass witnessing, remaking audiences as virtual witnesses and as vulnerable subjects. This mass production of audiences as subjects of virtual witnessing is further confounded today online where one may not be entirely clear what one is bearing witness to. Technologies of trauma coexist within a culture of trauma within modernity, illuminating modernity as an unsettled and unsettling epoch of incessant circulation of violence through the varied offerings of disasters, war, and carnage. The mass re-distribution of trauma via technologies produces audiences as witnessing subjects, and bearing witness distanced from its moral obligations leaves the ambit a contested terrain for humanity. The pervasive circulation and distribution of trauma through technologies and the production of witnessing subjects in tandem make vulnerability as constitutive of humanity, inscribing violence as an immanent aspect of modernity.
